# Dental utilization of active duty/previous active duty US military: a cross-sectional analysis of the 2010 Behavior and Risk Surveillance Survey

**DOI:** 10.13070/rs.en.1.888

**Published:** 2014

**Authors:** R Constance Wiener, Usha Sambamoorthi, Richard J Jurevic

**Affiliations:** 1 Dental Practice and Rural Health, School of Dentistry, Department of Epidemiology, School of Public Health, West Virginia University, USA; 2 Department of Pharmaceutical Systems and Policy, West Virginia University School of Pharmacy, Robert C. Byrd Health Sciences Center, USA; 3 Department of Oral Medicine, School of Dentistry, West Virginia University, USA

## Abstract

**Introduction:**

The purpose of this study is to understand dental utilization of 1) individuals serving/having served in active duty in the U.S. military as compared with the general public and 2) individuals who are currently serving as compared with individuals who are no longer active duty, but have been in active duty within the previous year.

**Methods:**

The Behavior and Risk Surveillance Survey, 2010, was used in cross-sectional analyses to determine the comparisons. Chi square and multivariable logistic regression analyses were applied.

**Results:**

70.7% of participants who had served/currently serving had a dental visit within the previous 12 months; 69.9% of the general public reported a dental visit (p = 0.0265). 69.8% of participants who had served/currently serving had a dental hygiene visit within the previous 12 months and 68.1% of the general public reported a dental hygiene visit (p <0.0001). The adjusted odds ratio (AOR) for participants who had served/currently serving vs. the general public was 1.10 (95% Confidence Interval [CI] 1.05, 1.16; p<0.0001) for dental visits and 1.11 (95%CI 1.05, 1.17; p<0.0001) for dental hygiene visits.

**Conclusion:**

Participants who are serving or have served were more likely to have any dental visit and dental hygiene visit than the general public; but the results were not substantively important.

Irregular dental visits affect quality of life and aspects of overall health. For example, 47.2% of Americans (64.7 million) have mild, moderate, or severe periodontal disease [1]. And periodontal disease has been associated with poorer diabetes control, cardiovascular disease, and a variety of other chronic inflammatory diseases. Regular dental visits have been recognized as an important component of individual medical readiness by the Department of Defense. In 2010, the United States had 1,431,000 military personnel on active duty [2]. Non-combat dental emergencies have a negative impact on military operations. A retrospective study of the dental emergency rates for Army personnel in Bosnia in 2000 indicated 156 dental emergencies per 1000 soldiers per year [3]. A study of all reported U. S. Army dental emergencies from 2009-2010 indicated 11,642 soldiers had dental emergencies, resulting in an average time loss of 24 hours per unit per week and an estimated financial cost in time of $14.7 million and an estimated cost in treatment of $13.9 million [4]. Additionally, there is a negative impact on combat effectiveness.4

The officials at the Department of Defense, being aware of the impact of non-combat dental emergencies in deployment, have Dental Readiness instructions within the instructions for Individual Medical Readiness. The instructions apply to the Army, Navy, Air Force, Marines, and Coast Guard (when the Coast Guard is not operating as a service within the Navy) [5] [6]. The minimum goal for the overall Individual Medical Readiness program is more than 75% of service members being fully medically ready [5]. The stated goal for dental readiness is 95% [6].

A Dental Fitness Class 1 or Class 2 rating (DFC) status is required for personnel to qualify for mobilization. There are 4 DFC ratings ranging from Class 1 in which an individual has a current examination (an examination within the previous 12 months) and no dental needs to Class 4 (DFC4) in which the person is not considered deployable [5] [6].

In Class 1 (DFC1), an individual has a current examination (an examination within the previous 12 months) and no dental needs. An individual with Class 1 designation are worldwide deployable [5] [6].

In Class 2, an individual has a current dental exam, and does require non-urgent care, but his or her dentist reports that the condition will remain stable and not become an urgent dental need during the next 12 months [5] [6]. An urgent dental need/emergency need is a condition of oral disease, trauma, loss of function, or other concern that causes a patient to seek immediate dental treatment.6 The non-urgent needs could include caries which are likely to remain stable for the next 12 months, repair of minor defective restorations, or a dental prophylaxis [5] [6]. This classification also includes treatment that can be deferred for 12 months for stable, advanced periodontal disease; temporomandibular disorder in remission; or symptomless unerupted/partially erupted/malposed teeth which are not pathological but are recommended for prophylactic removal, etc. [5] [6]. The instructions in this classification are that if a service man or woman has active orthodontic treatment, the treatment should be made passive if the deployment is less than 6 months; and if the deployment is greater than 6 months, there should be consideration of removal of active appliances and placing the dentition into passive retention [5] [6].

The Class 2 determination requires the examining dentist to predict if an urgent dental issue or a dental emergency situation is likely to occur. Civilian examining dentists from across the country are not calibrated in making such determinations; therefore, the reliability of the classification is questionable. Also, dental emergencies resulting from ulcerations, trauma, or referred pain of maxillary sinusitis are not predictable [7].

Another criticism of attempting to make a Class 2 determination is the lack of an analysis component for future risk [8]. Military personnel may not have an immediate acute need, but there may be a documented need for care at the Class 2 level which becomes emergent; or a person about to deploy may undergo extensive treatment before being deployed and he or she may develop emergent post-treatment problems [8]. Prediction of future dental need is difficult and an acceptable level of prediction has not been determined. In 1983, a study of deployment to the Sinai indicated a dental emergency rate of 160 dental emergencies/1000 soldiers/year [8]. In 2000, soldiers deployed to Bosnia had a dental emergency rate of 156/1000 soldiers/year (121 dental emergencies/1000 Dental Fitness Class 1 soldiers/year and 185 dental emergencies/1000 Dental Fitness Class 2 soldiers/year) [3]. Researchers in 2011, using a time frame of 3 ½ months and a sample of 3940 soldiers classified as deployable (Classes 1 and 2 combined, N=3806) and nondeployable (N=134) indicated that there were 39 dental emergencies in the nondeployable group (29.1%) and 216 in the deployable group (5.7%) [10]. The risk ratio was 5.13; 95%; Confidence interval: 3.82,6.88 [10]. These and similar studies indicate that although there may be a need for improvement in prediction, the dental emergencies for the military personnel being deployed have been similar across many years.

There is a need to develop a valid and reliable model for predicting dental emergencies which includes caries risk, periodontal health and tobacco use.8,11 There is also a need for standardization of the classifications to limit confusion and improve prediction [11].

In Class 3 (DFC3), an individual requires urgent or emergent dental care.5,6 He or she is not normally considered worldwide deployable [5] [6]. This classification includes needed follow-up treatment, treatment that cannot be maintained for 12 months, acute periodontal needs, radiographic pathosis that requires treatment, chronic infections, acute temporomandibular disorders, or edentulous areas requiring immediate treatment for communication, or mastication, or acceptable esthetics [5] [6].

In Class 4 (DFC4), an individual needs a periodic dental evaluation (the last dental visit was greater than the previous 12 months), or the individual has an unknown classification [5] [6]. He or she is not considered worldwide deployable [5] [6].

In the general population overall dental care, as measured by dental utilization, has been reported to be decreasing for adults since the mid-2000s [12]. In 2003, 42% of U.S. adults visited the dentist within the previous year, whereas in 2010, 37% of U.S. adults were reported to have visited the dentist [12]. A variety of reasons, such as dental anxiety [13] [14] [15] [16] [17] [18] [19], need for care [20], and financial barriers [21] [22] [23] [12] may explain infrequent dental visits or dental avoidance.

However, among individuals serving in the military or who have served in the military, there is a potential for a carry-over effect to have internalized the importance of the Dental Readiness instructions, to take charge of their oral situation, to plan, to use critical thinking, and to be less likely to leave things to chance. Therefore, it is highly plausible that dental utilization of individuals who are serving or who have served in active duty in the U.S. military may be substantially higher than individuals who have not served in the U.S. military. While there have been studies of dental utilization among the active military personnel, these studies have been specific to specific branches and have not examined carry-over effects.

Therefore, the objectives of this study are to better understand 1) dental utilization of individuals who are serving or who have served in active duty in the U.S. military as compared to the general public which had not served in the U.S. military and 2) dental utilization of individuals who are currently serving as compared with individuals who are no longer active duty, but have been in active duty within the previous year. The rationale for the study is that there is a need to know if returning personnel are more or less likely to utilize dental care and to inform policies to encourage dental utilization if needed.

The research hypotheses are: 1) there will be a statistically significant difference between the dental utilization and utilization of preventive services among individuals who have been active duty or who are currently active duty in the U.S. military vs. the general U.S. public which had never served in the U.S. military; and 2) dental utilization and utilization of preventive services among participants who are currently active duty will be equivalent to those who are not active duty but were active within the previous 12 months.

## Methods

The West Virginia Institutional Review Board approved this study (#1402191167). The researchers of this study used a cross-sectional design involving secondary data analysis of the 2010 Behavioral Risk Factor Surveillance System (BRFSS) survey, the most recent BRFSS data available with dental utilization and service status. The BRFSS is a telephone survey of the population of the adult (18 years and above) U.S. and its territories. BRFSS researchers used random-digit dialing to create a nationally representative sample. Cellular phones were not included in the 2010 survey. The survey has been conducted annually as a national surveillance since 1993 through the Centers for Disease Control and Prevention (CDC) and state health departments. It has a complex survey design. The researchers from the CDC developed probability samples of households using stratification. Weights were used to accommodate the differences in selection probability, non-response, and non-coverage. The design is detailed online at the BRFSS website. There were 451,075 records in the 2010 BRFSS survey. The BRFSS interviewers obtained informed consent from all individuals who were surveyed [24].

This study involved publicly available data. The sample was selected from participants with complete dental data concerning dental visits and complete service data. The final sample size was 422,143.

### Dependent variables: dental visit and dental hygiene visit

The primary outcome variable, dental visit, was the presence or absence of any dental visit within the past 12 months. It was measured using the BRFSS variable: adults that have visited a dentist, dental hygienist, or dental clinic within the past year. The secondary outcome variable, dental hygiene visit, was the presence or absence of a dental hygiene visit within the past 12 months. It was measured using the BRFSS question, “How long has it been since you had your teeth cleaned by a dentist or dental hygienist?” The researchers dichotomized the responses to: within the past year; or more than the past year.

### Key independent variables: military service categories

The key independent variable for the comparison of current/previous active service vs. no previous active service was determined by the researchers using the response to the question in the BRFSS survey: “Have you ever served on active duty in the United States Armed Forces, either in the regular military or in a National Guard or military reserve unit? Active duty does not include training for the Reserves or National Guard, but does include activation, for example, for the Persian Gulf War.” The responses were categorized to current/previous active service and no current/previous active service.

The key independent variable for the subgroup analysis for the comparison of participants who were currently active vs. participants who had been active during the past 12 months, but were not currently active, was determined by the researchers using the same BRFSS question while restricting the analyses to respondents who reported, “Yes, now on active duty,” or “Yes, on active duty during the past 12 months, but not now.”

### Other variables of interest

Other variables were included based upon the components of the Anderson Health Belief Model [25] [26]. These included predisposing factors: sex (female vs. male); race/ethnicity (Other vs. Non-Hispanic Whites); and age (18-40 years vs. 40 years and older); enabling factors: education (high school, some college/technical school, college/technical school degree and above vs. less than high school); and income ($15000-$24999, $25000-$34999, $35000-$49999, $50000 and above vs. less than $15000); need for care: cardiovascular disease (yes vs. no); and personal health practices: smoking status (current smoker, former smoker vs. never smoker); physical activity (participation in any physical activities or exercises outside of work, such as running, calisthenics, golf, gardening, or walking for exercise, yes vs. no); and obesity (overweight, obese vs. normal weight).

The researchers completed statistical analyses with SAS 9.3 (Carey, NC, USA). The analysis included frequency analyses, chi square analyses, and regression analyses which considered the weightings and complex design of the BRFSS in the analyses. The level of significance selected a priori was 0.05. The logistic regression was adjusted for the variables of interest and a 95% confidence interval was obtained from the model.

## Results

### Study sample

There were 422,143 participants in the sample to determine if there was a difference in dental visit utilization. There were 69.8% reporting a dental visit within the year. The sample had 62.5% females, 78.7% non-Hispanic whites, 82.8% 40 years and older, and 61.1% with an education beyond a high school degree. The income mode was $50,000 and above (37.1%). Details are presented in Table 1.

The study sample to determine if there was a difference in dental hygiene utilization was a subset of the 422,143 participants. It was a smaller sample since individuals who were edentulous were excluded. There were 385,001 in the dental hygiene sample. There were 64.5% reporting a dental hygiene visit within the year. The sample had 51.5% females, 69.6% non-Hispanic whites, 62.1% 40 years and older and 64.6% with an education beyond a high school degree. The income mode was $50,000 and above (51.8%).

### Comparisons of U.S. Military vs. General Public

The bivariate relationships between dental visits and dental hygiene visits within the past year and the variables of interest are presented in Table 2. There were 70.7% of participants who served or are currently serving and who had a dental visit within the previous 12 months as compared with 69.9% of individuals who had not served (p=0.0270). For a dental hygiene visit within the previous 12 months, the percentages were 69.8%, and 68.1% (p<.0001), respectively.

Statistical significance also occurred for dental visit with other variables: sex (72.0% of females and 67.8% of males); race/ethnicity (73.2% of non-Hispanic whites and 62.7% of Others); age (67.4% of participants18-40 years compared with 71.4% of 40 years and older); education (the higher the education level, the greater the number of people who had a dental visit within the previous 12 months); and income (the higher the income, the greater the number of people who had a dental visit within the previous 12 months).

Similarly, for dental hygiene visit within the previous 12 months, there were differences with the variables: sex (70.6% of females and 65.8% of males); race/ethnicity (71.9% of non-Hispanic whites and 60.0% of Others); age (64.0% of participants18-40 years compared with 70.8% of 40 years and older); education (the higher the education level, the greater the number of people who had a dental hygiene visit within the previous 12 months); income (the higher the income, the greater the number of people who had a dental hygiene visit within the previous 12 months).

In the multivariable, adjusted logistic regression on dental visits within the past year (Table 3), the participants who reported current or past active military service were more likely to have had a dental visit within the past year than the general public which had not served in the military. The adjusted odds ratio (AOR) was 1.10 (95% Confidence Interval [CI] 1.05, 1.16; p<0.0001).

In the multivariable, adjusted logistic regression on dental hygiene visits within the past year, the participants who reported current or past active military service were more likely to have had a dental hygiene visit within the past year than the general public which had not served in the military.

### Comparisons among active Military and not active Military personnel

Although the data are not presented in tabular form, there were 2634 participants who were currently active or had been active within the previous 12 months but were not currently active. Overall, 83.3% of the participants reported a dental visit within the year. This corresponded with 89.8% of currently active and 76.7% of active within the year but not currently active having had a dental visit within the year (a 13.1% difference). There were 2431 eligible participants for a dental hygiene visit (edentulous participants were excluded). Of the eligible population, 80.5% reported a dental hygiene visit within the previous year. There were 87.0% of currently active and 73.6% of the participants who were active within the year but who were not currently active who had a dental hygiene visit (a 13.4% difference).

In the multivariable, adjusted logistic regression on dental visits within the past year (Table 4), the participants who reported current active duty were more likely to have had a dental visit within the past year than participants who were active duty within the year, but not currently active. The adjusted odds ratio (AOR) was 2.74 (95% CI: 1.77, 4.23; p<0.0001). Other variables were also significant in the analysis.

In the multivariable, adjusted logistic regression on dental hygiene visits within the past year, the participants who reported current active duty were more likely to have had a dental hygiene visit within the past year than participants who were active duty within the year, but not currently active. The adjusted odds ratio (AOR) was 2.66 (95% CI: 1.77, 4.02; p<0.0001).

## Discussion

In this study, there were statistically significant, but negligible differences (1%) in any dental utilization and preventive dental care among individuals who are or were in the military vs. the general public which had not served in the military. Even after controlling for predisposing, enabling, need, and health care practices, those who are currently serving in the military were more likely to have any dental and any preventive dental care compared to not-active military and the general public.

However, among the people who have served within the year, participants who are currently active were more likely to have had a dental visit within the past year as compared with participants who had served in the military in the past 12 months but were not currently active. The differences were found in any dental utilization (89.8% vs. 76.7%) and any preventive dental care (87.0% vs. 73.6%). The Department of Defense policy requires active duty personnel to have annual dental examinations. Requirements exist and policies are in place for the people who are serving or may be called to serve to have annual dental examinations, however there continues to be less dental readiness in the Reserve and National Guard forces [6].

This research is useful in that it provides a surveillance of compliance with dental readiness preparedness using a nationally conducted survey. The data may be used to inform future policies to improve dental preparedness. From previous studies, it is known that there are fewer dental emergencies and disease non-battle injuries among the active duty personnel who were deployed without dental disease [6]. Many successful health promotion programs and attempts to improve dental readiness have, nevertheless, met obstacles such as poor dental IQ, lack of affordable access to care and dental fear. Millions of dollars are used to address dental readiness, but a “just in time” dental readiness persists as an ongoing issue and unnecessary distraction [6]. In 2008, extensive efforts were made however 7-15% of personnel arrived at the federal mobilization platform in Class 3 status [28].

Proposals to improve dental readiness include funding medical days, bonuses for attaining or maintaining dental readiness, insurance plans to pay for dental readiness, requiring recruits to have needed dental care through a program such as the First Term Dental Readiness program, providing vouchers to have needed dental work completed, providing dental treatment for demobilizing personnel, using the Department of Veteran Affairs dental benefit, and similar programs [6]. Individual responsibility for oral health care, improving the perception of the need for oral health care for both the commander and the service personnel, oral health education, self-care for daily oral infection control, and having the person fully understand his or her role or responsibility for maintaining readiness [6] [28].

In this study, there was a higher number of dental visits for both the participants who served or are currently serving in the U.S. military (70.7%) and the general public which had not served (69.9%) as compared with a recent study of 2000-2010 data from the Medical Expenditure Panel Survey, which is a complex, national telephone survey of household in the US with components for insurance/employer and medical provider. The researchers, who used the Medical Expenditure Panel, indicated 37% of adults had a dental visit within the past 12 months [12]. The Medical Expenditure Panel data is from people who have made visits using Medicaid or dental insurance, and the data is not collected from people who are private payers. Nevertheless, since the current study used telephone interviews without verification of the self-report, the survey may be subject to a social desirability bias resulting in an increased report of dental visits. Another limitation of this study was that cell phones were not included in the survey, which may have resulted in potential respondents being excluded from the sample. When a cross-sectional study design is used, it is not possible to draw causal inferences, however the researchers may use the study to highlight areas of future study: Are military personnel who were exposed to combat more or less likely to continue having a high prevalence of dental visits, particularly considering the impact of traumatic brain injury (TBI), post-traumatic stress disorder, and similar conditions with dental anxiety and dental avoidance? (One recent study’s data indicate that Veteran visits decrease with serious mental illnesses). Does the decrease in dental care utilization correlate with other emergent care visits such as respiratory infections and/or odontogenic infections? TBI and increased incidence of swallowing disorders and dysphagia has not been well investigated, and may be associated with recurrent pulmonary infections and aspiration pneumonia. Is dental anxiety different between personnel who have served or are serving in the military and the general population? Several factors may increase dental utilization by the military personnel-- the hierarchical military order, with the need to follow orders; the provision of dental care at no cost; and the access to care. Do these factors influence continued dental care later in life? What are the dental care problems observed in recent veterans currently being treated within the Veterans Affairs (VA) hospital system? Although dental care is available in the VA system, it is based upon level of disability and thus may impact moderately injured veterans more than veterans with 100% disability.

## Conclusion

Participants who are serving or have served in the past 12 months had a negligible but statistically significant greater dental utilization compared with participants who have never served. Currently active participants had higher utilization rates compared to participants who were active within the year. There continues to be a need to improve the level of dental utilization and preventive services.

## Figures and Tables

**Table 1:** Sample Characteristics, BRFSS, 2010.

	Did not serve in US military		Served in US military		Currently active US military		Not Active now, but had been within the previous 12 months	
	Number	wt%	Number	wt %	Number	wt %	Number	wt %
Sex								
Female	259211	70.9	4595	8.1	253	19.0	366	28.1
Male	106220	29.1	52115	91.9	1080	81.0	935	71.9

Race/ethnicity								
Non-Hispanic white	284624	77.9	47535	83.8	954	71.6	974	74.9
Other	76382	20.9	8236	14.5	361	27.1	312	24.0

Age								
18 to less than 40	65954	18.1	3273	5.8	704	52.8	298	22.9
40 and above	296461	81.1	53093	93.6	620	46.5	996	76.6

Education								
Less than high school	35742	9.8	3253	5.7	26	2.0	91	7.0
High school graduate 107776	29.5 16372	28.9	286	21.5	389	30.0		
Some college/technical	96013	26.3	16661	29.4	436	32.7	385	29.6
HE graduate & above	124996	34.2	20309	35.8	582	43.7	433	33.3

Income								
Less than $15000	39715	10.9	3868	6.8	23	1.7	106	8.2
$15000-$24999	56581	15.5	8689	15.3	96	7.2	172	13.2
$25000-$34999	37080	10.2	6999	12.3	106	8.0	148	11.4
$35000-$49999	46332	12.7	9322	16.4	226	17.0	165	12.7
$50000 and above	1343773	36.9	21932	38.7	785	58.9	520	40.0

Smoking Status								
Current smoker	56745	15.5	8869	15.6	188	14.1	256	19.7
Former smoker	100586	27.5	27863	49.1	364	27.3	406	31.2
Never smoker	205788	56.3	19655	34.7	770	57.8	634	48.7

Diabetes								
Diabetes/Pre/Gest	52558	14.4	11296	19.9	78	5.9	189	14.5
No Diabetes	312548	85.5	45359	80.0	1255	94.2 1109	85.2	

Physical Activity								
Yes	265531	72.7	41937	74.0	1177	88.3	1008	77.5
No	99478	27.2	14678	25.9	155	11.6	292	22.4

Body Mass Index								
Normal	126062	34.5	14956	26.4	404	30.3	387	29.8
Overweight	122591	33.6	25424	44.8	660	49.5	540	41.5
Obese	99693	27.3	15523	27.7	245	18.4	338	26.0

Cardiovascular disease								
Yes	30252	8.3	11039	19.5	58	4.4	161	12.4
No	331794	90.8 44953	79.3	953	71.5 1124	86.4		

Dental visit within year								
Yes	255586	69.9	38939	68.7	1197	89.8	998	76.7
No	109847	30.1	17771	31.3	136	10.2	303	23.3

Hygiene visit within year^1^								
Yes	232875	69.6	34609	69.0	1129	87.0	893	73.6
No	101955	30.5	25562	31.0	169	13.0	321	26.4

wt%: weighted column percentage; HE: Higher education; Gest-gestational.

1 For those with teeth.

**Table 2:** Sample Characteristics by Dental Visits and Dental Hygiene Visits, BRFSS 2010.

	Dental Visit	Wt%	p-value	Hygiene Visit	Wt%	p-value
Served or Currently serving in the US Military			0.0265			<.0001

Yes	38939	70.7		34609	69.8	
No	255586	69.9		232875	68.1	

Sex			<.0001			<.0001

Female	187078	72.0		170308	70.6	
Male	107447	67.8		97176	65.8	

Race/ethnicity			<.0001			<.0001

Non-Hispanic white	239096	73.2		219112	71.9	
Other	52001	62.7		45382	60.0	

Age			<.0001			<.0001

18-40 years	47297	67.4		43977	64.0	
40 and older	244652	71.4		221156	70.8	

Education			<.0001			<.0001

Less than high school	16386	47.3		12123	45.2	
High school	76687	61.8		66270	59.5	
Less than college/tech	79399	61.8		72239	67.6	
College/tech/above	121379	82.1		116280	80.3	

Income			<.0001			<.0001

Less than $15000	18973	46.2		14009	41.8	
$15000-$24999	35086	52.3		28693	49.0	
$25000-$34999	28639	62.8		25327	60.6	
$35000-$49999	40501	69.4		37213	67.0	
$50000 and above	132304	82.7		127335	80.9	

Smoking status			<.0001			<.0001

Current smoker	34953	54.4		28667	50.4	
Former smoker	89876	71.0		80841	70.3	
Never smoker	167898	74.1		156452	72.4	

Diabetes			<.0001			<.0001

Diabetes/Pre/Gestational	38878	62.0		33408	61.7	
No	255430	71.0		233887	69.1	

Physical Activity			<.0001			<.0001

Yes	228368	73.8		211422	72.0	
No	65854	58.0		55806	56.0	

Body Mass Index (BMI)			<.0001			<.0001

Normal	103806	74.2		95559	72.9	
Over weight	105482	71.0		96215	69.5	
Obese	73307	63.9		64918	61.4	

Cardiovascular disease			<.0001			<.0001

Yes	23902	59.8		19796	60.8	
No	268416	70.8		245916	68.8	

Chi Square test (Rao-Scott Chi-Square); Wt % indicates weighted percentage of individuals who attended a dental or dental hygiene visit weighted to account for complex sample design. Consequentially, dividing cell size by total sample will not result in weighed percentage; Tech-technical school.

**Table 3:** Unadjusted and Adjusted Odds Ratios and 95% Confidence Intervals from Logistic Regression on Presence/Absence of Dental visits and Dental Hygiene visits, BRFSS, 2010.

	Dental Visit			Dental Hygiene Visit		
	OR	95% CI	p-value	OR	95% CI	p-value
Unadjusted Serving/served in Active Military yes v no	1.04	1.01, 1.08	0.0275	1.08	1.04, 1.13	<.0001

Adjusted^1^ Serving/served in Active Military yes v no	1.10	1.05, 1.16	<.0001	1.11	1.05, 1.17	<.0001

1 Adjusted for sex (female vs male), race/ethnicity (Other vs Non-Hispanic White), age (18-39 years vs 40 and older), education (High School, some college/technical school, degree and above vs less than high school), income ($15,000-24,999, $25,000-34,999, $35,000-49,999, $50,000 and above vs less than $15,000), smoking status (current, former vs never), physical activity (yes vs no), diabetes (yes vs no), cardiovascular disease (yes vs no), and body mass index (overweight, obese vs normal weight). OR: odds ratio.

**Table 4:** Unadjusted and Adjusted Odds Ratios and 95% Confidence Intervals from Logistic Regression on Presence/Absence of Dental visits and Dental Hygiene visits for Active Military v Active within the previous 12 months, BRFSS, 2010.

	Dental Visit			Dental Hygiene Visit		
	OR	95% CI	p-value	OR	95% CI	p-value
Unadjusted Active duty now v Not active now but was within the year	3.53	2.43, 5.13	<.0001	3.13	2.22, 4.40	<.0001

Adjusted^1^ Active duty now v Not active now but was within the year	2.74	1.77, 4.23	<.0001	2.66	1.77, 4.02	<.0001

1 Adjusted for sex (female vs male), race/ethnicity (Other vs Non-Hispanic White), age (18-39 years vs 40 and older), education (High School, some college/technical school, degree and above vs less than high school), income ($15,000-24,999, $25,000-34,999, $35,000-49,999, $50,000 and above vs less than $15,000), smoking status (current, former vs never), physical activity (yes vs no), diabetes (yes vs no), cardiovascular disease (yes vs no), and body mass index (overweight, obese vs normal weight). OR: odds ratio.
